# Avortements clandestins compliqués et médicaments de la rue à Brazzaville

**DOI:** 10.11604/pamj.2020.36.143.18816

**Published:** 2020-06-30

**Authors:** Darius Eryx Mbou Essie, Hermann Ndinga, Ange Niama, Guy Oyere, Grace Kifoueni, Jean-Rosaire Ibara

**Affiliations:** 1 Faculté des Sciences de la Santé, Université Marien Ngouabi, Brazzaville, République du Congo,; 2 Service de Gynécologie-Obstétrique, Hôpital de Référence de Talangaï, Brazzaville, République du Congo,; 3 Direction des Soins de Santé Primaires, Ministère de la Santé, Brazzaville, République du Congo

**Keywords:** Morbi-mortalité maternelle, avortement clandestin, médicaments de la rue, Maternal morbi-mortality, clandestine abortion, street drugs

## Abstract

**Introduction:**

les avortements clandestins augmentent la morbi-mortalité maternelle en Afrique sub-Saharienne et sont favorisés par les législations restrictives et la faiblesse prévalence contraceptive. A Brazzaville l´automédication par les « médicaments de la rue » semble être un procédé abortif fréquent. L´étude visait à déterminer la proportion des « médicaments de la rue » et le profil sociodémographique des patientes impliquées.

**Méthodes:**

nous avons réalisé une étude longitudinale de juillet à décembre 2018, 67 patientes admises pour complications d´avortement provoqués ont été recrutées à l´hôpital de Talangaï. Leurs (i) caractéristiques socio démographiques, (ii) obstétricales et (iii) les caractéristiques de l´avortement (procédé, implication du manganguiste, rang et coût de l´avortement) ont été collecltées et analysées avec le logiciel Epi info 7. Nous avons comparé les moyennes avec le test de student, les proportions avec le CHI-2, p était fixé à <0,05.

**Résultats:**

l´âge moyen était de 25 ans ±6,6 ; 59,7% étaient au collège, 53,8% sans activité génératrice de revenu dans et 38,8% des cas vivaient seules dans; 15% avaient une paternité refusée. Les « médicaments de la rue » étaient retrouvés dans 74,5%, où le coût moyen de l´avortement était 3500 CFA (7$US) et 29500CFA (59$US) chez les professionnels de santé. Les enquêtées du lycée étaient plus nombreuses (73,69%) à connaitre au moins méthode contraceptive (p<0,05). Les célibataires (p=0,000) et celles qui connaissaient une méthode contraceptive (p=0,003) exprimaient plus l´intention d´adopter une contraception. **Conclusion:** lutter contre les « médicaments de la rue » ainsi que sécuriser l´avortement volontaire sont nécessaires pour limiter les avortements clandestins compliqués.

## Abstract (en)

**Introduction:**

clandestine abortions increase maternal morbi-mortality in sub-Saharan Africa and are closely linked to restrictive legislation and low contraceptive prevalence. In Brazzaville street drugs are commonly used to induce abortion. The purpose of this study is to determine street drug prevalence and socio-demographic characteristics of these patients.

**Methods:**

we conducted a longitudinal study of 67 patients with induced abortion complications admitted to the Talangaï Hospital from July to December 2018. (i) Socio-demographic (ii) and obstetrical (iii) characteristics as well as abortion features (procedure, Manganguiste involvement, abortion rank and cost) were collected and analyzed using EPI info 7 software. We compared the means using student’s test, proportions with CHI-2, p value was set to < 0.05.

**Results:**

the average age of patients was 25 years ± 6.6; 59.7% of them were attending college, 53.8% had no income-generating activity, 38.8% lived alone and in 15% of cases biological father had denied paternity. Street drugs had been used in 74.5% of cases, mean abortion cost was 3500 CFA (US$7) and 29500CFA (US$59) when it had been performed by health-care professionals. High school respondents were more likely (73.69%) to know at least contraceptive methods (p<0.05). Greater numbers of singles (p=0.000) and of those who knew a contraceptive method (p=0.003) expressed the intention to use contraception. **Conclusion:** combatting the use of street drugs and securing the right to safe voluntary abortion are necessary to limit complications due to clandestine abortions.

## Introduction

Les complications des avortements clandestins sont responsables jusqu´à 30% des décès maternels en Afrique [1,2] et au Congo en particulier où la mortalité maternelle s´élève à 426/100.00 naissances vivantes [3]. Les législations restrictives et répressives [4], la faible prévalence contraceptive [5], les normes sociales « procréatrices » [6], l´expansion des médicaments de la rue [7-10] sont autant de facteurs sous tendant ces avortements clandestins en Afrique. L´automédication favorisée par la vente illicite des substances médicamenteuses dites « médicaments de la rue » pourrait devenir un procédé fréquemment utilisé aux fins abortives. A Brazzaville ces « médicaments de la rue » sont proposés par des fournisseurs illégaux désignées par le vocable « Manganguistes ». Cette étude visait à montrer l´ampleur des « médicaments de la rue » vendus à but abortif par ces fournisseurs illégaux parmi les avortements clandestins compliqués reçus dans un hôpital de référence de Brazzaville. Spécifiquement il s´est agi de décrire le profil social des enquêtées et les caractéristiques des avortements clandestins compliqués.

## Méthodes

Nous avons réalisé une étude longitudinale à visée descriptive de Juillet à Décembre 2018 (6 mois) à la maternité de l´hôpital de référence de Talangaï, maternité de référence de 5 districts sanitaires de Brazzaville (Talangaï, Djiri, Ouenzé, Poto-poto et Moungali). Les avortements provoqués volontairement [11] ne sont pas autorisés au Congo, tout avortement provoqué est un avortement clandestin (art 317 code pénal, art 8 code de déontologie des professions sociales et de la santé). L´échantillonnage a été un recrutement exhaustif de toutes les patientes consentantes admises pour avortement clandestin compliqué pendant la période de l´étude; les avortements provoqués par intoxication médicamenteuse accidentelle, les patientes en détresse vitale ou décédées ont été exclues. Les données ont été récoltées par interview à l´aide d´un questionnaire non standardisé adapté sur 10 patientes, administré par l´investigateur. Les variables étudiées ont été: (i) socio-démographiques: nationalité, âge, niveau d´instruction, activité génératrice de revenu, statut marital, acceptation de la paternité; (ii) obstétriques: nombre de grossesse, nombre d´enfants vivants, âge de la grossesse avortée; (iii) caractéristiques de l´avortement: rang, méthode utilisée, personne ayant conseillé la méthode, lieu d´acquisition des substances abortives, coût; et (iv) la connaissance et l´attitude face à la contraception (citer une méthode contraceptive et exprimer le besoin de contraception à la sortie de l´hôpital). La statistique descriptive a été faite par le calcul de la moyenne, de la médiane et l´écart type pour les variables quantitatives et les fréquences pour les variables qualitatives. La comparaison des moyennes a été faite par le test t de student; celles des fréquences entre deux modalités d´une variable qualitative s´est faite par le test de Chi carré (Chi-2) de Pearson ou le test exact de Fisher en cas d´effectif inférieur à 5. Le seuil de significativité était fixé à p<0,05. Les données ont été saisies et codées dans le logiciel Epi info version 7.

**Ethique:** l´accord du comité national d´éthique a été obtenu, ainsi que l´autorisation du médecin chef du district sanitaire de Talangaï. Le consentement éclairé verbal des patients a été recherché avant l´entretien. Les résultats de cette enquête ont été transmis aux autorités sanitaires pour mesures correctrices.

## Résultats

Au total 67 patientes ont été inclues; les caractéristiques socio-démographiques sont détaillées dans le Tableau 1. L´âge moyen était de 25,2 ans ±6,6; 59,7% avaient un niveau scolaire du collège et 29,8% du lycée. Les enquêtées exerçaient une activité génératrice de revenu dans 53,8% des cas. Elles cohabitaient avec le conjoint dans 38,8% des cas et avaient un refus de paternité de la grossesse avortée dans 15% des cas. Pour les caractéristiques obstétricales. La moyenne de grossesse par enquêtée était de 3,3±2, extrêmes 1 et 9; le mode était à 3. La moyenne d´enfant a été de 1,7 ± 1,7, extrême de 0 à 8. Les caractéristiques de l´avortement sont données par le Tableau 2: le rang de l´avortement en cours était premier chez 59,7% (40/67) des enquêtées, deuxième chez 26,9%, troisième chez 10,4%, cinquième et septième chez 1,5% respectivement. La moyenne d´avortement pour l´ensemble était de 1,6 ± 1, la moyenne chez les enquêtées vivant avec leur conjoint a été de 1,5 et celles qui vivaient seules de 1,7 différence non significative (t=0,88, p= 0,3, IC 95% [-0.2606; 0.6753]). Concernant l´âge de la grossesse avortée, 27,7% des enquêtées n´en connaissant pas l´âge, 43% avaient une grossesse de 6 à 12 semaines et les 29,3% autres une grossesse de 13 à 24 semaines.

**Tableau 1:** caractéristiques socio-démographiques des enquêtées

	n	%
**Nationalité**		
Congolaise	63	94
Autres	4	6
**Age**		
[16-25]	32	48
[25-34]	27	40
[34-43]	8	12
**Niveau scolaire**		
Sans ou primaire	2	3
Collège et lycée	60	89,5
Universitaire	5	7,5
**Vit avec un conjoint**		
Oui	26	38,8
Non	41	61,2
**AGR**		
Oui	30	53,8
Non	35	46,2
**Paternité**		
Accepté	57	85
Refusé	10	15

**Tableau 2:** caractéristiques liées à l´avortement

	n	%
**Méthode utilisée**		
Comprimé ou tisane	53	79,1
Curetage/aspiration	8	12
Injection	6	8,9
**Personne ayant conseillé la méthode**		
Moi-même ou mon conjoint	47	70,2
Ami ou un parent	9	13,4
“ Manganguiste ”	11	16,4
**Lieu d´acquisition ou d´exécution**		
Cabinet médical	13	19,4
Marché ou chez un “ Manganguiste ”	50	74,6
Pharmacie	1	1,5
Maison d´un agent de santé	3	4,5

Dans 79,1% elles ont avorté à l´aide des comprimés ou tisanes acquis dans 74,6% chez le « Manganguiste » exerçant en solitaire ou dans un marché où il a été directement consulté dans 16,4% et 20,9% chez les personnels de santé par procédé médical ou aspiration endo utérine. Le coût du procédé de l´avortement était connu chez 95,5% (64/67). Il variait de 100 à 10.000 francs CFA avec un prix moyen de 3700 CFA pour les comprimés ou la tisane acheté chez les « Manganguiste ». Pour les enquêtées qui ont été avortées par un agent de santé dans son cabinet ou maison d´habitation le prix variait de 12.000 à 60.000 francs CFA avec un prix moyen d´environ 29500 FCFA. Le mode d´avortement n´était pas lié au niveau scolaire (p=0,1) ni à l´exercice d´une activité génératrice de revenu (p=0,4). Pour la connaissance et l´attitude face à la contraception. Seules 52,4% des enquêtées connaissaient au moins une méthode contraceptive et cette proportion était plus importantes (73,69%) chez les enquêtées qui avaient le niveau d´instruction du lycée (p<0,05). Les méthodes citées étaient: le préservatifs (19 fois), la pilule (11 fois), le respect du cycle menstruel (3 fois) et les injectables (2 fois). Les enquêtées ont exprimé le besoin de contraception dans 76,9%, cette proportion a été plus importante chez celles qui avaient cité au moins une méthode contraceptive (p=0,01) et celles qui vivaient seules (p=0,000) comme le montre le Tableau 3.

**Tableau 3:** besoin de contraception en fonction du statut marital et de la connaissance de méthode contraceptive

	Besoin de contraception		p
**Vivre avec son conjoint**	**Oui**	**Non**	**Total 0,000a**
Oui	14	12	26
Non	37	4	41
Total	51	16	67
**Connaissance de méthode contraceptive**			**0,01b**
Oui	31	4	35
Non	20	12	32
Total	51	16	67

a très significatif, b significatif

## Discussion

La taille de l´échantillon est certes relativement faible, mais cette étude a eu le mérite d´alerter sur l´impact des « médicaments de la rue » dans les avortements clandestins. La moyenne d´avortement chez nos enquêtées qui reposait sur la seule déclaration a pu aussi être sous-estimé car avorter est perçu comme un comportement déviant en Afrique [12,13]. L´anonymat du questionnaire et le respect du secret professionnel visait à minimiser ce biais. L´avortement est une pratique très répandue dans le monde [4,14] et en Afrique avec un taux de 36% et où 13% des grossesses se terminent en avortement provoqué. Ce phénomène est plus fréquent dans les pays où la législation est répressive contre l´avortement [2,15]. La tranche d´âge 16-34 ans a été majoritaire chez nous (88%), proche des 82,3% rapporté en Zambie [16]; c´est la période de forte activité génitale. Les motifs d´avortement n´ont pas été explorés dans notre étude. La moyenne retrouvée n´était pas différente selon que la femme vivait seule ou non (1,7 vs 1,5; t=0,8, p=0,3), qu´elles avaient une activité génératrice de revenu ou non (1,4 vs 1,6; t=-0,9, p=0,3). Les enquêtées qui cohabitaient avec un conjoint donc plus à risque de grossesse non programmée étaient les plus nombreuses à ne pas exprimer le besoin de contraception (p=0,000). Cette réticence peut être liée à l´obstacle que représentent parfois les avis des conjoints. Une étude en RDC rapporte 29% de refus de contraception pour ce motif [17].

Les médicaments de la rue fournis par le « Manganguiste » étaient retrouvé dans près de 80% des avortements dont une proportion non négligeable (16,4%) incitée par lui-même; ceci indépendamment du niveau scolaire ou de l´exercice d´une activité génératrice des revenus. La composition et l´efficacité réelles des substances délivrées par le « Manganguiste » restent à prouver, comme dans les études de Kachi et Koffi [7,18] le type et l´indication des molécules abortives ne sont pas aussi retrouvées. Ce qui traduit le côté clandestin des ventes pour ce but et le détournement des indications classiques. La vulgarisation et la disponibilité croissante de certains puissants abortifs comme le misoprostol [19] peut laisser suspecter sa présence dans les compositions médicamenteuses vendues. Le faible coût (8 fois moins) du procédé chez le « Manganguiste » explique en général la préférence des médicaments de la rue par rapport au circuit officiel du médicament [7,10]. De même la clandestinité dans laquelle le « Manganguiste » exerce illégalement la pharmacie et la médecine représente un gage de discrétion pour les enquêtées. La légalisation de l´IVG en France visait à briser cette clandestinité qui favorise les avortements non sûrs responsables de la morbi-mortalité maternelle [20]. Selon une étude sur les trajectoires empruntées pour les avortements provoqués en Zambie où la loi est plus permissive de l´avortement provoqué [21,22], 16,1% et 20,5% impliquaient respectivement des médicaments abortifs et des substances traditionnelles acquis par des voies non médicales [16].

L´association illégalité et insécurité de l´avortement peut être remise en débat [23] mais à notre sens l´avortement par implication des « Manganguiste » reste à risque grave car ils ignorent les complications de leurs produits et n´offrent pas d´assistance en cas complications abortives. Au Congo l´autorisation de la promotion contraceptive a été très tardive en 2005 (Loi Zoula, Assemblée Nationale, R Congo), le système de santé doit fournir des efforts importants pour améliorer la prévalence contraceptive globale qui n´est que 20% en general et 3% pour la pilule [5]. La conférence internationale sur la population a recommandé le conseil à la planification familiale comme soins post abortifs pour limiter les avortements répétés (Nations Unies, 1994, « rapport de la conférence internationale sur la population et le développement). Nous avons ainsi évalué la perception des enquêtées sur la contraception planification. Notre étude a montré que plus de la moitié des enquêtées ne connaissaient pas une méthode contraceptive, pourtant les deux tiers ont en exprimé l´intention d´adoption: tant d´occasions manquées. Aussi longtemps que perdura cette « ignorance » de la contraception, les besoins non couverts en contraceptifs et leur corolaires les grossesses non programmées et les avortements auront une prévalence croissante. Une vraie aubaine pour les « Manganguiste » qui apparaissent comme la réponse à la demande d´avortement dans ces environnements très répressifs, où l’avortement côtoie l´expansion des « médicaments de la rue » [7,18]. Comme le montre notre étude, presque 3 enquêtées sur 5 ont acquis les substances abortives dans un marché ou une boutique.

## Conclusion

Cette enquête préliminaire a montré que la prise des « médicaments de la rue » à but abortif a été le mode de l´avortement clandestin compliqué; le « Manganguiste » y est impliqué en délivrant les substances après les avoir conseillées. Des efforts de promotion de la contraception et de lutte contre le phénomène des « médicaments de la rue » sont nécessaires. En perspective une enquête rétrospective multicentrique comparative sur l´avortement clandestin en milieu hospitalier versus « médicaments de la rue » serait une contribution au débat sur la « sécurisation » de l´avortement volontaire au Congo.

### Etat des connaissances sur le sujet

- Les avortements clandestins sont plus fréquents dans les pays où tout avortement est interdit et puni;
- Ils entrainent souvent des complications graves et Impliquent les personnels de santé plus ou moins qualifiés;
- Les « médicaments de la rue » sont un danger pour la santé publique.

### Contribution de notre étude à la connaissance

- Notre étude a montré que les vendeurs des médicaments de la rue deviennent le moyen le plus fréquemment utilisé pour l´avortement;
- Lutter contre les vendeurs de médicaments de la rue est une approche efficace pour réduire la morbi mortalité maternelle en Afrique sub-Saharienne.

## Remerciements

Nous remercions: le médecin chef du District Sanitaire de Talangaï pour avoir autorisé cette enquête, l´équipe de tous les médecins du service de la maternité de l´Hôpital de Talangaï et toutes les patientes qui ont accepté de répondre au questionnaire.
